# Delayed coronary artery stenosis: a rare complication of the left atrial clipping device

**DOI:** 10.1093/icvts/ivad183

**Published:** 2023-11-08

**Authors:** Yuki Imamura, Ryosuke Kowatari, Tomonori Kawamura, Hiroaki Ichikawa

**Affiliations:** Department of Thoracic and Cardiovascular Surgery, Hirosaki University School of Medicine, Hirosaki, Japan; Department of Thoracic and Cardiovascular Surgery, Hirosaki University School of Medicine, Hirosaki, Japan; Department of Thoracic and Cardiovascular Surgery, Hirosaki University School of Medicine, Hirosaki, Japan; Department of Cardiology, Hirosaki University School of Medicine, Hirosaki, Japan

**Keywords:** Atrial appendage, Surgery, Coronary stenoses

## Abstract

A clipping device may impinge on the coronary artery following left atrial appendage occlusion during cardiac surgery, causing rare cardiac ischaemia perioperatively. This report highlights a case of delayed severe coronary artery stenosis resulting in ventricular fibrillation 2 months after cardiac surgery with the implantation of a left atrial clipping device. Following a percutaneous coronary intervention, the patient underwent clip removal surgery. Postoperative three-dimensional heart model verification revealed that the base of the left atrial appendage was more dorsal than usual, thereby increasing the potential risk of the clip impinging on the coronary artery. We should remember that this rare complication can occur after left atrial clipping, either in the early postoperative period or later.

## INTRODUCTION

Using a clipping device for left atrial appendage (LAA) occlusion is safe and effective for patients with atrial fibrillation during cardiac surgery [1]. Although rare, the clipping device may inadvertently impinge on the coronary artery, causing serious cardiac ischaemia during the perioperative period [2, 3]. We report a case of coronary artery stenosis resulting in ventricular fibrillation 2 months after clipping device implantation, confirmed by postoperative three-dimensional heart model verification.

## CASE REPORT

**Figure 1:**
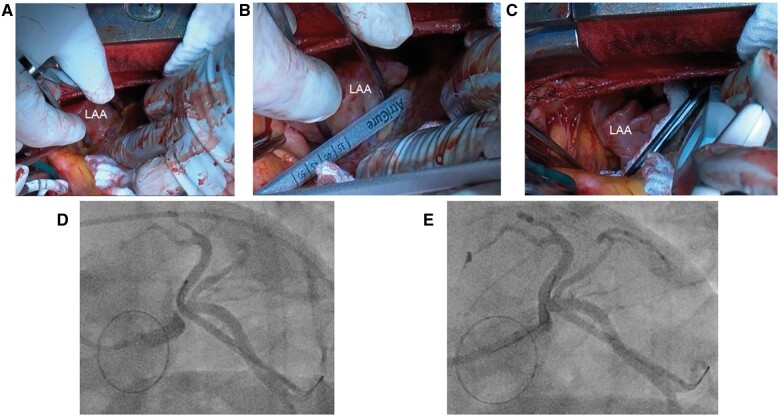
Intraoperative images showing the (**A**) left atrial appendage sizing before (**B**) and after clip placement (**C**). Coronary angiograms before (**D**) and after (**E**) percutaneous coronary intervention.

A 32-year-old male with a unicuspid aortic valve underwent surgery due to severe aortic stenosis and atrial fibrillation. After median sternotomy, a 40-mm AtriClip device (AtriCure, West Chester, OH, USA) was implanted under cardiac arrest (Fig. 1A–C). Aortic valve replacement was performed using an SJM Mechanical Heart Valve (St. Jude Medical Inc., Minneapolis, MN, USA). Although the patient was discharged 14 days after the procedure without complications, he was readmitted to our hospital for ventricular fibrillation 2 months after discharge.

**Figure 2:**
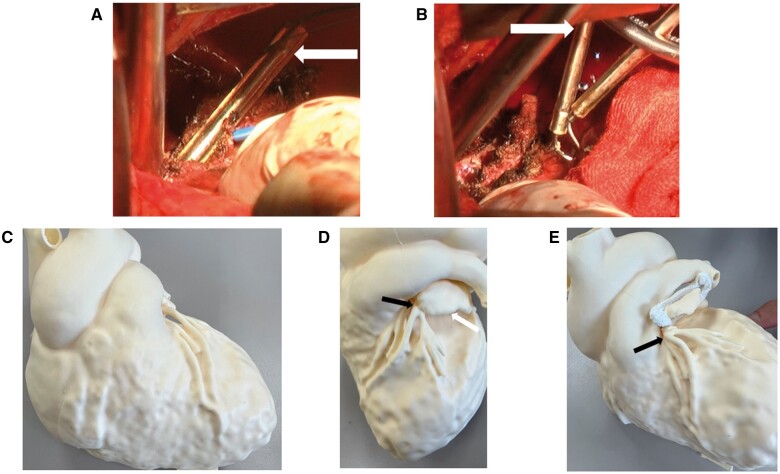
Intraoperative images showing the cutting of the clip’s posterior side (white arrow) using a wire cutter (**A**) and its removal (**B**). The 3D heart model of this case shows the front (**C**) and lateral views (black arrow: left main coronary artery, white arrow: left atrial appendage). A clip tackling the left main coronary artery (**D** and **E**).

Coronary angiography revealed severe left main coronary artery (LMCA) stenosis due to its proximity to the anterior side of the AtriClip (Fig. 1D). The patient underwent percutaneous coronary intervention (PCI) (Fig. 1E) and required intensive care for 3 weeks. An additional 3 weeks were required for an improvement of his general condition. The AtriClip device was surgically removed 2 months after PCI. The adhesion between the LMCA and AtriClip was severe. The posterior side of the clip was cut using a steel wire cutter; this resulted in the removal of the clip without damaging the LAA (Fig. 2A and B). A 4/0 polypropylene running suture was positioned at the base of the appendage. The patient was discharged 15 days after surgery without complication. Written informed consent was obtained from the patient for the publication of this report.

## DISCUSSION AND CONCLUSION

Our experience highlights the rare but lethal midterm complications associated with atrial clipping devices. Only 3 coronary artery occlusion cases have been reported. Two occurred during the operation, and 1 occurred within 24 h postoperatively [2, 3]. It appears unlikely that ischaemia would occur after a 2-month delay, as seen in this case. The risk of compromise during implantation is minimized owing to the excellent visibility of the anatomical structures. However, implanting a clipping device under cardiac arrest necessitates caution, as the operator may position it too deep into the base of the LAA. In the present case, the postoperative three-dimensional heart model verification (Fig. 2C) showed that the LMCA and base of the LAA passed through the dorsal side further than usual (Fig. 2D). This anatomical feature could pose a potential risk when employing a clipping device (Fig. 2E). Additionally, adhesions are fibrotic connections resulting from tissue trauma and ischaemia during surgery [4] that can lead to late LMCA stenosis, as is the present case. We believe that intermittent stimulation of the clip causes myocardial ischaemia, and further investigation is necessary. We removed the clip to avoid stent injury in the LMCA, despite the absence of ischaemic symptoms after PCI. We should remember that this rare complication can occur after surgery using an atrial clipping device, either in the early postoperative period or later. Preoperative computed tomography could characterize the relationship between the coronary artery and the LAA when considering a clipping device.

In conclusion, when the base of the LAA is more dorsal than usual, care should be taken when using a clipping device to avoid mid-term coronary artery stenosis.

**Conflict of interest:** none declared.

## Data Availability

The data of this study are available from the corresponding author upon reasonable request. Interdisciplinary CardioVascular and Thoracic Surgery thanks Ahmet Baris Durukan, Luca Di Marco, and the other anonymous reviewer(s) for their contribution to the peer review process of this article.
